# *PITX2C* increases the stemness features of hepatocellular carcinoma cells by up-regulating key developmental factors in liver progenitor

**DOI:** 10.1186/s13046-022-02424-z

**Published:** 2022-06-28

**Authors:** Lingxi Jiang, Xia Wang, Fangfang Ma, Xuelong Wang, Minmin Shi, Qian Yan, Ming Liu, Juan Chen, Chaoran Shi, Xin-yuan Guan

**Affiliations:** 1 Department of General Surgery, Ruijin Hospital, Shanghai Jiao Tong University School of Medicine, 200025 Shanghai, People’s Republic of China; 2 Department of Clinical Oncology, Li Ka Shing Faculty of Medicine, The University of Hong Kong, Room L10-56, Laboratory Block, 21 Sassoon Road, Pokfulam, Hong Kong, China; 3 Research Institute of Pancreatic Diseases, Shanghai Jiao Tong University School of Medicine, Shanghai, China; 4 State Key Laboratory of Liver Research, The University of Hong Kong, Hong Kong, China; 5 Department of Colorectal Surgery, Guangdong Institute of Gastroenterology, Guangdong Provincial Key Laboratory of Colorectal and Pelvic Floor Diseases, The Sixth Affiliated Hospital, Sun Yat-sen University, Guangzhou, China; 6 Affiliated Cancer Hospital and Institute of Guangzhou Medical University, Guangzhou Municipal and Guangdong Provincial Key Laboratory of Protein Modification and Degradation, School of Basic Medical Sciences, Guangzhou Medical University, Guangzhou, China

**Keywords:** PITX2A/B/C, HCC, Tumorigenicity, Stemness, Developmental factors in liver progenitor

## Abstract

**Background:**

Tumor cells exhibited phenotypic and molecular characteristics similar to their lineage progenitor cells. Liver developmental signaling pathways are showed to be associated with HCC development and oncogenesis. The similarities of expression profiling between liver progenitors (LPs) and HCC suggest that understanding the molecular mechanism during liver development could provide insights into HCC.

**Methods:**

To profile the dynamic gene expression during liver development, cells from an in vitro liver differentiation model and two paired hepatocellular carcinoma (HCC) samples were analyzed using deep RNA sequencing. The expression levels of selected genes were analyzed by qRT-PCR. Moreover, the role of a key transcription factor, pituitary homeobox 2 (*PITX2*), was characterized via in vitro and *vivo* functional assays. Furthermore, molecular mechanism studies were performed to unveil how *PITX2C* regulate the key developmental factors in LPs, thereby increasing the stemness of HCC.

**Results:**

*PITX2* was found to exhibit a similar expression pattern to specific markers of LPs. *PITX2* consists of three isoforms (*PITX2A/B/C*). The expression of *PITX2* is associated with tumor size and overall survival rate, whereas only *PITX2C* expression is associated with AFP and differentiation in clinical patients. PITX2A/B/C has distinct functions in HCC tumorigenicity. PITX2C promotes HCC metastasis, self-renewal and chemoresistance. Molecular mechanism studies showed that PITX2C could up-regulate RALYL which could enhance HCC stemness via the TGF-β pathway. Furthermore, ChIP assays confirmed the role of PITX2C in regulating key developmental factors in LP.

**Conclusion:**

PITX2C is a newly discovered transcription factor involved in hepatic differentiation and could increase HCC stemness by upregulating key transcriptional factors related to liver development.

**Supplementary Information:**

The online version contains supplementary material available at 10.1186/s13046-022-02424-z.

## Background

Tumor cells are widely recognized to exhibit phenotypic similarity with lineage progenitor cells. Hepatocellular carcinoma (HCC) is one of the most common solid tumors worldwide with an inferior prognosis [1]. Compared to normal liver tissues, HCC typically expresses markers of liver progenitors (LP) at high levels, including alpha-fetoprotein (AFP), cytokeratin 7(CK7), and cytokeratin 19 (CK19), which are often associated with poor outcome [2, 3]. A variety of growth factors, such as FGF, BMP, HGF, Wnt and TGF-β promote liver progenitor cell migration, proliferation and survival [4]. The Wnt/β-catenin and TGF-β signaling pathway promotes hepatocyte and biliary epithelial cell differentiation during hepatic maturation [5, 6]. These signaling pathways also appear to enhance HCC stemness [7–9]. Evidence suggested that the key factors governing hepatic differentiation in LPs are of critical importance in HCC tumorigenicity and progression.

In our previous study, an in vitro liver differentiation model from human embryonic stem cells (ES) was established [10]. ES could generate definitive endoderm cells (DE) by treating the culture medium with a certain concentration of Activin. Then, the addition of FGF and BMP induced the differentiation of DE differentiate into LPs, which expanded and matured after treatment with a combination of HGF, OSM and dexamethasone. In the last stage of this model, premature hepatocytes (PHs) were acquired [4]. Combining the deep RNA sequencing of cells at four stages with two paired HCC clinical samples transcriptomic data, we screened a group of genes that were actively expressed in LPs and had higher expression levels in HCC than non-tumor tissue. Among these genes, a transcription factor, pituitary homeobox 2 (PITX2) was of interest due to its location in the central of gene regulatory network (Pathway Common) [11], with a unique expression pattern significantly associated with HCC differentiation and poor outcome.

PITX2, a member of the bicoid/paired-like homeobox gene family, is a multifunctional transcription factor [12–14]. PITX2 consists of six transcript variants that are translated into three isoforms: *PITX2-V16* (PITX2A), *PITX2-V245* (PITX2B), *PITX2-V3* (PITX2C). PITX2A/B/C is produced by alternative splicing and transcription [15]. Compared to *PITX2A* and *PITX2B*, *PITX2C* uses an alternative promoter [16, 17]. All isoforms contain dissimilar amino terminus, identical homeodomain and C-terminal domains. Three isoforms differentially regulate the transcription of the target genes. *PITX2C* is the dominant isoform in developing and adult left atrium [18]. The roles of PITX2 seem to be controversial in cancer research. On the one hand, lower levels of PITX2 expression have been reported in patients with breast cancer, prostate cancer and colon cancer as well as being associated with poor prognosis [19–22]. On the other hand, PITX2 has also been identified as a potential oncogene in thyroid cancer, prostate cancer and ovarian cancer [20, 23–25]. The isoforms of PITX2 may possess diverse functions in tumorigenicity.

In the present study, the functions of the three isoforms of PITX2 were characterized in HCC. The mRNA level of *PITX2C* was associated with AFP and differentiation in HCC clinical samples. *PITX2C* overexpression increased the stemness-related characteristics of HCC in in vitro and in vivo functional assays, while mechanistic studies revealed that *PITX2C* rather than *PITX2A/B* could activate TGF-β signaling by regulating the transcription of *RALYL*. Furthermore, PITX2C was found to regulate and cooperate with key factors related to hepatic differentiation and HCC tumorigenicity.

## Materials and methods

### HCC samples and cell lines

Two cohorts of patients with HCC were carefully evaluated histologically and included in this study. One cohort for checking the mRNA expression level by qRT-PCR included 93 pairs of primary HCC tissues and their matched non-tumor tissues collected after surgical resection at Sun Yat-sen University Cancer Center (Guangzhou, China) between 2001 and 2005 (Cohort-1). The surgical specimens (both tumor and adjacent non-tumor tissue) were processed immediately after the operation and snap-frozen in liquid nitrogen for RNA extraction. The other cohort for pathological study of tissue microarray (TMA) is a retrospective cohort of 132 patients with HCC who also underwent hepatectomy at Sun Yat-sen University Cancer Center (Guangzhou, China) between 2001 and 2008 (Cohort-2). The dissected tumor tissues were embedded in paraffin block and used for tissue microarray (TMA) construction. None of these patients were diagnosed with autoimmune diseases and received chemotherapy or radiotherapy before operation. The mean age of these enrolled HCC patients was 48 years (range, 21–79 y). The median follow-up period was 34.5 months (range, 6–96.3 mo). Over 85% of these patients had hepatitis B virus infection. About 20% of patients had cirrhosis. Moreover, the clinical characteristics of these patients included age, sex, serum AFP, serum HBsAg, differentiation, tumor size, metastatic status [26]. The status of differentiation was determined by histopathologists according to the pathological properties of tumor tissues [27]. Differentiation (I-II) was defined as well-differentiated tumor tissues, whereas differentiation (III-IV) was defined as poorly-differentiated ones. The samples used in this study were approved by the committees for Ethical Review of Research Involving Human Subjects at the Sun Yat-Sen University Cancer Center. Human immortalized hepatic cell lines, MiHA, LO2, and HCC cell lines (i.e. MHCC97L, H2P, PLC-8024, SNU-475, SNU-449, Hep3B and HepG2) were tested for mycoplasma contamination. STR DNA profiling analysis was conducted for cell line authentication. Details about these cell lines was found in our previous study [26, 28].

### In vitro and *vivo* functional assays

In vitro and *vivo* functional assays was reported in our previous study [26]. A detailed description could be found in the Supplementary Materials and Methods.

### Immunohistochemical staining (IHC) and antibodies

IHC staining was performed according to the standard procedure. Details could be found in Supplementary Materials and Methods. The antibodies used for IHC included PITX2 from Sigma-Aldrich (St. Louis, MO), proliferating cell nuclear antigen (PCNA) from Immunoway Biotechnology Company (Plano, TX, USA), and c-Myc, NANOG, CD133 and EPCAM from Cell signaling Technology (Danvers, MA, USA).

### Luciferase reporter assays

The pGL3.0 luciferase expression system was purchased from Promega Corporation (Australia) and was performed according to the manufacturer’s instruction.

### Chromatin immunoprecipitation

A chromatin immunoprecipitation (ChIP) assay was performed as previously described [29]. Antibodies for ChIP included FLAG and H3K27Ac from Cell Signaling Technology (Danvers, MA, USA). The ChIP products were amplified using specific primers (Supplementary Table 1A). Details were described in Supplementary Materials and Methods.

### Statistical analysis

Statistical analysis was conducted using the SPSS software (version 17.0; SPSS, Inc., Chicago, IL, USA). Pearson’s Chi-square test was used to analyze the association of *PITX2* expression with clinicopathological parameters as well as *RALYL* expression. Kaplan-Meier plots and log-rank tests were used for survival analysis. Data are presented as the mean ± SD of three independent experiments. A *P* value less than 0.05 was considered statistically significant.

## Results

### Identification of PITX2C in an in vitro hepatocyte differentiation model

**Fig. 1:**
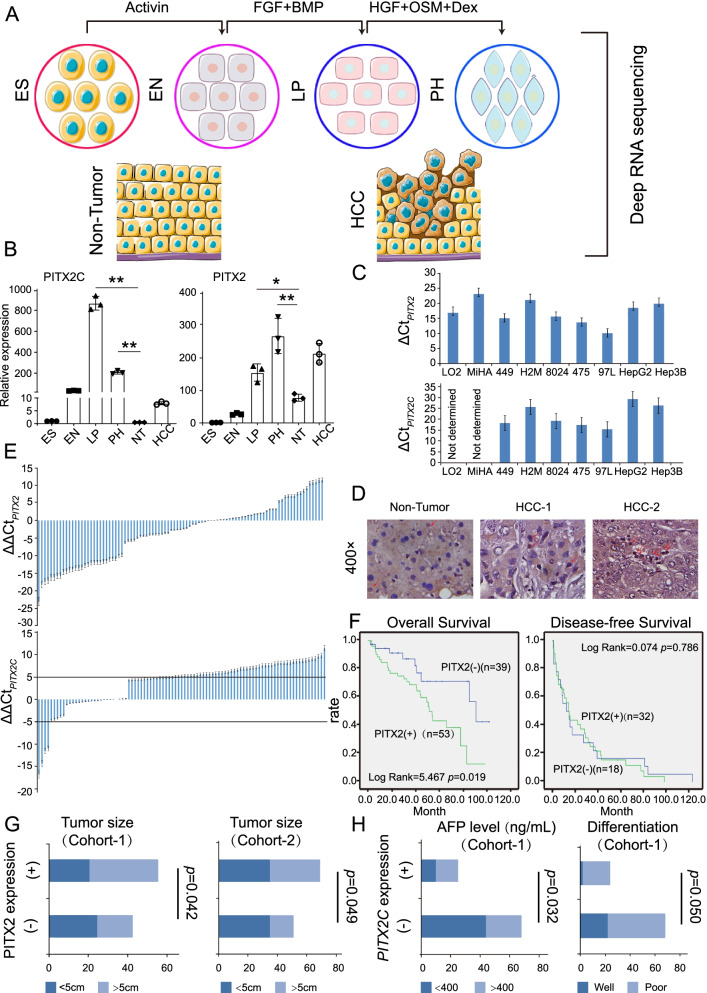
The expression and clinical significance of PITX2. **A** Establishment of an in vitro hepatocyte differentiation model, which induced human embryonic stem (ES) cells into definitive endoderm (DE), liver progenitor cells (LP) and premature hepatocytes (PH). Cells in the four stages of in vitro hepatocyte differentiation model together with two HCC and two adjacent non-tumor tissues were used for deep RNA sequencing **(B)** The relative expression level of *PITX2* and *PITX2C* in each stage of the in vitro hepatocyte differentiation model was confirmed using qRT-PCR. (*P < 0.05, **P < 0.01, independent Student’s *t*-test) **(C)** Bar chart of delta Ct (Top: ΔCt_*PITX2*_ = Ct_*PITX2*_-Ct_*GAPDH*_; Bottom: ΔCt_*PITX2C*_ = Ct_*PITX2C*_-Ct_*GAPDH*_) in two immortalized liver cell lines and seven HCC cell lines. *GAPDH* was used as a reference gene. The higher the ΔCt, the lower expression level of target gene. **D** Representatives of IHC staining images with anti-PITX2 in non-tumor, HCC with moderate PITX2 expression, HCC with high expression (400× magnification). Red arrows indicate the PITX2+ cells. **E** The relative expression level of *PITX2* in HCC clinical samples (Cohort-1) compared to the corresponding non-tumor tissue (Top). ΔΔCt _*PITX2*_ = (Ct _*PITX2 in tumor*_ – Ct _*GAPDH in tumor*_)-(Ct _*PITX2 in non-tumor*_ –Ct _*GAPDH in non-tumor*_). As 2^-ΔΔCt *PITX2*^ indicates the fold change of PITX2 in tumor relative to the corresponding non-tumor, PITX2 was up-regulated in 53 out of 93 HCC samples with ΔΔCt _*PITX2*_ < 0. The relative expression level of *PITX2C* in HCC clinical samples were evaluated by ΔΔCt _*PITX2C*_ = (Ct _*PITX2C in tumor*_ – Ct _*GAPDH in tumor*_)-20 as the *PITX2C* expression was almost absent in non-tumor tissue (Bottom). Five HCC cases showing stable expression of PITX2C as ΔΔCt _*PITX2C*_ < − 5. Two groups with distinct *PITX2C* expression: group (+) with ΔΔCt _*PITX2C*_ < 0; group (−) with ΔΔCt_*PITX2C*_ > 0. **F** Kaplan–Meier overall (left) and disease-free survival curve (right) of two HCC groups: PITX2 (+), patients with higher PITX2 expression; PITX2 (−), patients with lower PITX2 expression. **G** Association of PITX2 expression with tumor size in two HCC cohorts. PITX2 expression was determined by qRT-PCR or IHC. **H** Association of *PITX2C* expression with AFP and differentiation in HCC cohort-1. *PITX2C* expression is defined as positive in HCC samples when ΔΔCt _*PITX2C*_ < 0. **G-H** The X-axis represents the number of HCC cases in each group. The *P* value was calculated using Pearson Chi-square test

An in vitro hepatocyte differentiation model was previously established from ES cells into DE, LP and PH cells [26]. Transcriptome sequencing was performed to identify the gene profiling of four stages in the *vitro* hepatocyte differentiation model. The heatmap of the expression profiles together with the qRT-PCR results have demonstrated that the marker genes were highly specifically expressed in their corresponding stages [10, 26]. Thereafter, the expression pattern of four hepatic developmental stages (ES, EN, LP and PH) and two paired HCC clinical samples was analyzed (Fig. 1A). To identify the key factors involved hepatic differentiation and maturation regulation, a group of genes encoding nuclear proteins that are specifically expressed in the LP and PH stages were screened (Supplementary Fig.1A). Pathway Commons was used to analyze the regulatory and interaction networks between selected nuclear proteins. As a result, we found that PITX2 was located at the center of the network (Supplementary Fig.1B). Full-length of *PITX2A/B/C* contained varying 5′ sequences (Supplementary Fig.1C). Specific forward primers were designed to detect the six transcript variants, which were translated into three isoforms (*PITX2-V16*: PITX2A; *PITX2-V245*: PITX2B; *PITX2-V3*: PITX2C) (Supplmentary Table 1). The expression patterns of the three *PITX2* isoforms in the hepatic differentiation model were further confirmed by qRT-PCR. Surprisingly, only *PITX2C* showed its peak expression in the LP and PH stages and decreased in non-tumor tissues, as well as HCC tissues (Fig. 1B).

### Clinical significance of PITX2 in HCC

The expression levels of PITX2 in immortalized liver and HCC cell lines were examined using qRT-PCR and western blotting. These results indicated that PITX2 was expressed at higher levels in 97 L, PLC-8024, SNU-475 and SNU-449 cells (Supplementary Fig.1D). However, compared to *PITX2A* and *PITX2B*, lower mRNA levels of *PITX2C* was detected in cell lines, especially *PITX2C* was almost absent in two immortalized liver cells (LO2 and MiHA) (Fig. 1C). The protein levels of all PITX2 isoforms in a cohort of 132 HCC cases were examined by IHC staining. Higher levels of expression PITX2 were detected in 50.76% (66/132) of HCC tissues (Fig. 1D). Up-regulation of all transcript variants of *PITX2* at mRNA level was also confirmed in 56.9% (53/93) of HCC tissues by qRT-PCR, compared with adjacent non-tumor tissues. However, *PITX2C* was rarely expressed, as the values ofΔCt (Ct _*PITX2C in tumor*_ – Ct _*GAPDH in tumor*_) were over 25 in most of HCC cases. Only five cases showed stable expression of PITX2C (Fig. 1E). Specific *PITX2C* expression was further determined through Fluorescence in situ hybridization (FISH) staining of HCC and their adjacent non-tumor tissues. Similarly, the transcript of PITX2C was rarely detected in non-tumor tissue (Supplementary Fig. 1E). Kaplan-Meier survival analysis showed that higher levels of PITX2 expression were significantly associated with poorer overall survival (OS), but not with disease-free survival (DFS) rates (Fig. 1F). Similar results were observed in the TCGA database (Supplementary Fig. 1F). The clinical pathologic study revealed that PITX2 higher expression was significantly associated with tumor size in two in-house HCC cohorts (Fig. 1G). Interestingly, the mRNA level of *PITX2C* was associated with a relatively high AFP level and differentiation (Fig. 1H). These findings suggest that although PITX2 may contribute to the tumorigenicity of HCC, PITX2C plays a crucial role in regulating HCC differentiation and stemness.

### *PITX2A/B/C* has distinct functions in the tumorigenicity of HCC

**Fig. 2:**
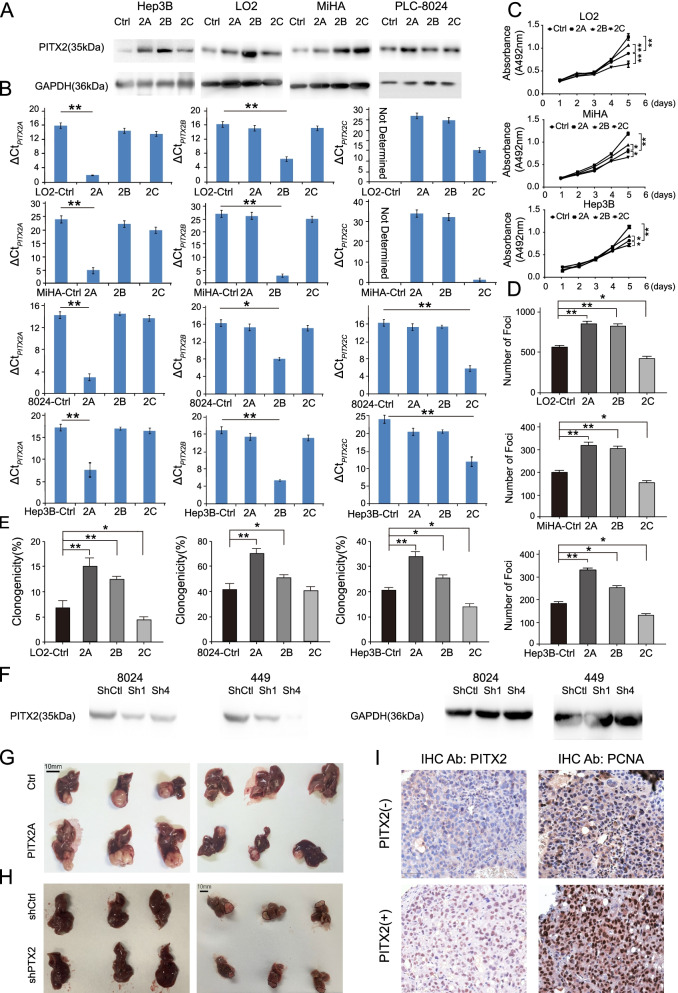
PITX2A/B/C has distinct functions in the tumorigenicity of HCC. **A** Western blotting analysis confirmed the ectopic expression of PITX2A/B/C in stable *PITX2A, 2B, 2C*-transfected LO2, MiHA, Hep3B and PLC-8024 cell, respectively. **B** The mRNA levels of PITX2A/B/C were detected by qRT-PCR using specific primers in stable *PITX2A, 2B, 2C*-transfected LO2, MiHA, Hep3B and PLC-8024 cell. ΔCt_*PITX2A*_ = Ct_*PITX2A*_-Ct_*GAPDH*_; ΔCt_*PITX2B*_ = Ct_*PITX2B*_-Ct_*GAPDH*_; ΔCt_*PITX2C*_ = Ct_*PITX2C*_ -Ct_*GAPDH*_. The higher the ΔCt, the lower expression level of target gene. **C** Cell proliferation between *PITX2A, 2B, 2C*-transfected cells and control cells was compared by XTT assay. **D** Quantitative analyses of foci numbers of each transfected group are shown in bar chart. **E** Quantitative analyses of colony numbers of each transfected group are shown in bar chart. **B-E** The results are expressed as mean ± SD of three independent experiments (*P < 0.05, **P < 0.01, independent Student’s *t*-test). **F** Two shRNAs targeting PITX2 (shPITX2–1 and shPITX2–4) could effectively decrease PITX2 expression in protein level detected by western blotting. shCtl was used as negative control. Representative images of excised orthotopic tumor formed by intrahepatic implantation experiment using *PITX2A-*transfected LO2 or MiHA cells and control cells **(G)** or shPITX2 and shCtl transfected PLC-8024 cells **(H)**. **I** Representative images of PCNA IHC staining in orthotopic tumors generated from cells with PITX2 higher expression and their corresponding control cells (400×, magnification)

To investigate the roles of PITX2A/B/C isoforms in tumorigenicity, the full-length sequences of the three isoforms were cloned into lentiviral vectors and stably transfected into MiHA, LO2, PLC-8024 and Hep3B cells. The ectopic expression of *PITX2A/B/C* was examined at protein level (Fig. 2A). The qRT-PCR results confirmed that the mRNA levels of the three isoforms were also enhanced in the corresponding transfected cells (Fig. 2B). XTT assays showed that the overexpression of *PITX2A* and *PITX2B* promoted cell proliferation. In contrast, *PITX2C* overexpression suppressed cell proliferation compared to that in controls (Fig. 2C). Similar results were obtained in foci formation assays and the colony formation capacity in soft agar (Fig. 2D, E and Supplementary Fig. 2A, B). Moreover, *PITX2* expression was knocked down in PLC-8024 and SNU-449 cells with two short hairpin RNAs (shRNAs) (Fig. 2F and Supplementary Fig.2C). As expected, *PITX2* knockdown significantly decreased cell proliferation, foci formation and colony formation in soft agar (Supplementary Fig.2D, E, F).

To further confirm whether PITX2 could promote HCC tumorigenicity in vivo, subcutaneous tumors induced by *PITX2A*-transfected LO2, MiHA and Hep3B cells and control cells were implanted into the livers of nude mice. The results showed that tumors induced by *PITX2A*-transfected cells were significantly larger (Fig. 2G, Supplementary Fig.2G-H). In addition, tumor volume was significantly decreased in the *PITX2* knockdown system (Fig. 2H and Supplementary Fig.2G). Moreover, IHC staining was performed for the orthotropic tumors. A stronger intensity of PCNA was found in tumors induced by higher *PITX2*-expressing cells (Fig. 2I). Taken together, these data suggest that PITX2A/B promotes cell proliferation and tumor growth in HCC, while PITX2C suppresses cell growth.

### *PITX2C* promotes cell mobility and self-renewal of HCC

**Fig. 3:**
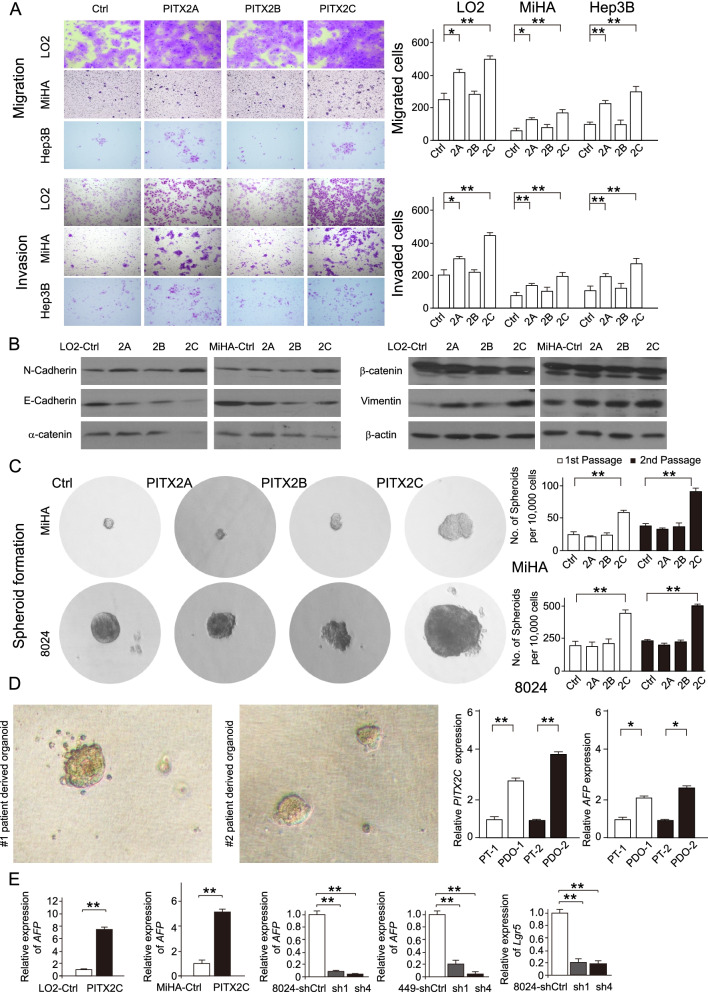
PITX2C promotes cell mobility and self-renew of HCC. **A** Representative images (left) and bar chart (right) of cell migration and invasion abilities in LO2, MiHA and Hep3B cells with *PITX2A, 2B, 2C*-transfection by Transwell (50,000 cells) and Matrigel invasion (100,000 cells) assays. Migrated and invaded cells were stained with crystal violet and counted under a microscope. Values indicate the mean ± SD of three independent experiments (*P < 0.05; **P < 0.01; independent Student *t* test). **B** Western blotting analysis was used to compare expressions of epithelial and mesenchymal markers between *PITX2A, 2B, 2C*-transfected cells and control cells. β-actin was used as loading control. **C** Spheroid formation assay was used to evaluate the self-renewal ability of *PITX2A, 2B, 2C*-transfected and control cells (left). The numbers of primary and secondary spheroids are calculated in the bar chart (right). Values indicate the mean ± SD of three independent experiments (*P < 0.05, **P < 0.01, independent Student’s *t*-test). **D** Representative images of organoids derived from two HCC patients. The mRNA levels of *PITX2C* and *AFP* were validated by qRT-PCR. PDO. Patient derived organoid; PT. primary tumor tissue. PT is used as control. Data are presented as the mean ± SD of 3 independent experiments (*P < 0.05, **P < 0.01, independent Student’s *t*-test). **E** Relative expression of *AFP* and *Lgr5* in *PITX2C* and *shPITX2*- transfected cells (Right: non-transfected cells were used as controls). Data are presented as the mean ± SD of 3 independent experiments

The effect of PITX2A/B/C on cell motility was characterized by cell migration and invasion. Transwell and cell invasion assays indicated that PITX2A/C significantly enhanced cell motility in both LO2, MiHA and Hep3B cells (Fig. 3A). EMT-related markers were also upregulated or downregulated in *PITX2C*-transfected cells, which further confirmed that *PITX2C* could promote cell mobility (Fig. 3B). *PITX2* knockdown significantly inhibited HCC cell motility to a lesser extent (Supplementary Fig. 3A).

As *PITX2C* is actively expressed in LPs and PH which usually exhibit enhanced self-renewal ability, we speculated that PITX2C may regulate HCC differentiation. *PITX2C* overexpression was found to markedly increase both the primary and secondary spheroid formation capacities in MiHA and PLC-8024, however, no significant difference was observed between *PITX2A/B* overexpressing cells and controls (Fig. 3C). Conversely, silencing *PITX2* decreased the size and number of spheroids formed in the PLC-8024 cells (Supplementary Fig.3B). The mRNA level of *PITX2C* was higher in HCC patient-derived organoids than in the corresponding primary tumor tissues (Fig. 3D). Moreover, qRT-PCR revealed that the fold change of two markers of LPs (*AFP* and *Lgr5*) were increased in *PITX2C* overexpressing cells but reduced after *PITX2* knockdown (Fig. 3E, Supplementary Fig.3E).

### *PITX2C* enhances the chemoresistance of HCC cells in vitro and in vivo

**Fig. 4:**
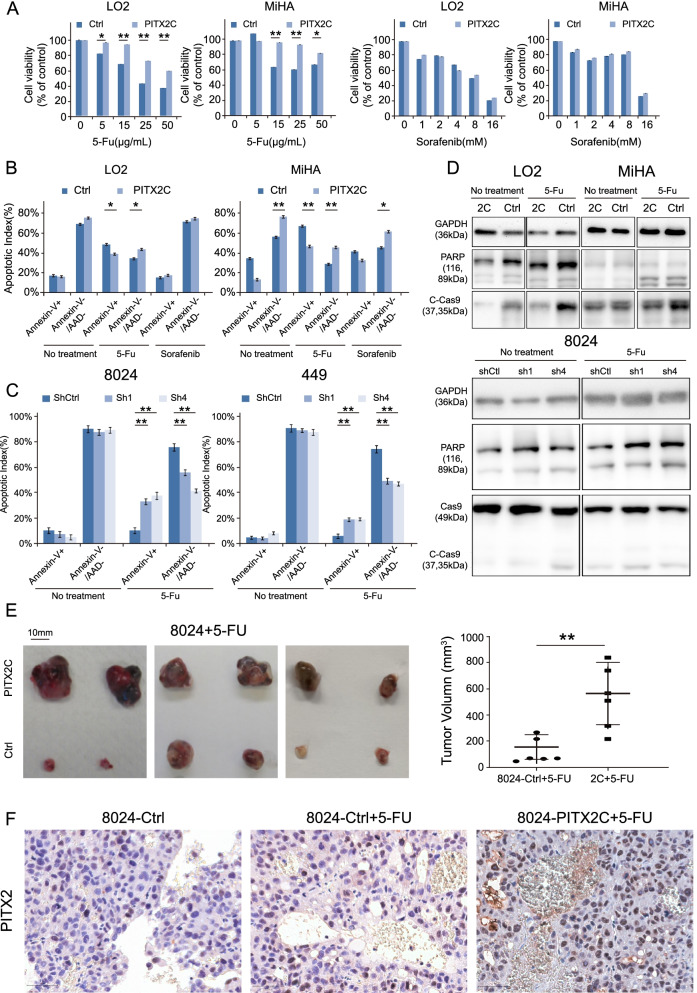
*PITX2C* enhances the chemoresistance of HCC cells. **A** Cell viabilities of *PITX2C*- and vector-transfected cells were detected by XTT assay after treatment with 5-Fu and Sorafenib at the indicated concentration for 48 h. The apoptotic indexes of vector-, *PITX2C*-transfected **(B)** and shCtl- and shPITX2-transfected cells **(C)** were detected by fluorescence-activated cell sorting-based Annexin V/AAD double staining after treatment with 5-Fu or Sorafenib at the indicated concentration for 48 h. **A-C** Values indicate the mean ± SD of three independent experiments with three repeats (*P < 0.05, **P < 0.01, independent Student’s *t*-test). **D** The activation of caspase-9, poly (ADP ribose) polymerase were compared between *PITX2C*- transfected cells (top),shPITX2-transfected cells (bottom) and control cells after 5-Fu treatment for 48 h. GAPDH was used as a loading control. **E** Subcutaneous tumors induced by indicated cells were treated with 5-Fu. The dose of 5 mg/kg 5-Fu could effectively shrink tumour size in control cells compared to *PITX2C*-transfected cells. The final tumor volumes are summarized in dot chart. The average tumour volume was expressed as the mean ± SD. *P* values were calculated using independent Student’s *t* test. **F** Representative IHC images show that PITX2 positive cells were enriched in 5-Fu treated cells (400×, magnification)

Chemoresistance is a stemness-related characteristic of tumor cells. Drug resistance allows tumor cells to survive in conventional or targeted therapies, ultimately leading to relapse [30]. The combination of cisplatin, alpha-interferon, doxorubicin and 5-flurouracil (5-Fu) yielded a promising overall response rate in a phase II trial [31]. As sorafenib is a standard first-line treatment for advanced HCC, clinical trials of sorafenib in combination with conventional chemotherapies in HCC patients are being conducted worldwide [32]. After treatment with 5-Fu at different concentrations, the cell viability of *PITX2C*-overexpressing cells was significantly higher than that of the controls (Fig. 4A). Consistently, the flow cytometry results revealed that the apoptotic index was lower in *PITX2C*-overexpressing cells than in the control cells (Fig. 4B, Supplementary Fig. 3C). In addition, the typical molecular indicators of apoptosis, namely caspase-9 and poly (ADP ribose) polymerase were markedly decreased in *PITX2C*-transfected cells compared with control cells (Fig. 4D). As expected, *PITX2* knockdown had the opposite effect (Fig. 4C-D and Supplementary Fig. 3D). However, PITX2C did not contribute to sorafenib resistance in HCC cells (Fig. 4A-B), implying that PITX2C may not regulate the targets of sorafenib, including VEGFR, PDGFR and RAF family kinases.

To further confirm the role of PITX2C in the chemoresistance of HCC in vivo, nude mice with xenograph tumors formed by *PITX2*C- and control-transfected PLC-8024 cells were treated with 5-Fu (50 mg/kg body weight) regularly. When the subcutaneous tumor reached a similar size of approximately 5 mm in diameter, 5-Fu was administrated via intraperitoneal injection every 5 days. Tumors induced by *PITX2C*-transfected cells grew faster and were larger than those in the control group (Fig. 4E). Intriguingly, *PITX2*-expressing cells were significantly enriched in tumors treated with 5-Fu (Fig. 4F, Supplementary Fig. 4).

### *PITX2C* enhances stemness by up-regulating key developmental factors in liver progenitor

**Fig. 5:**
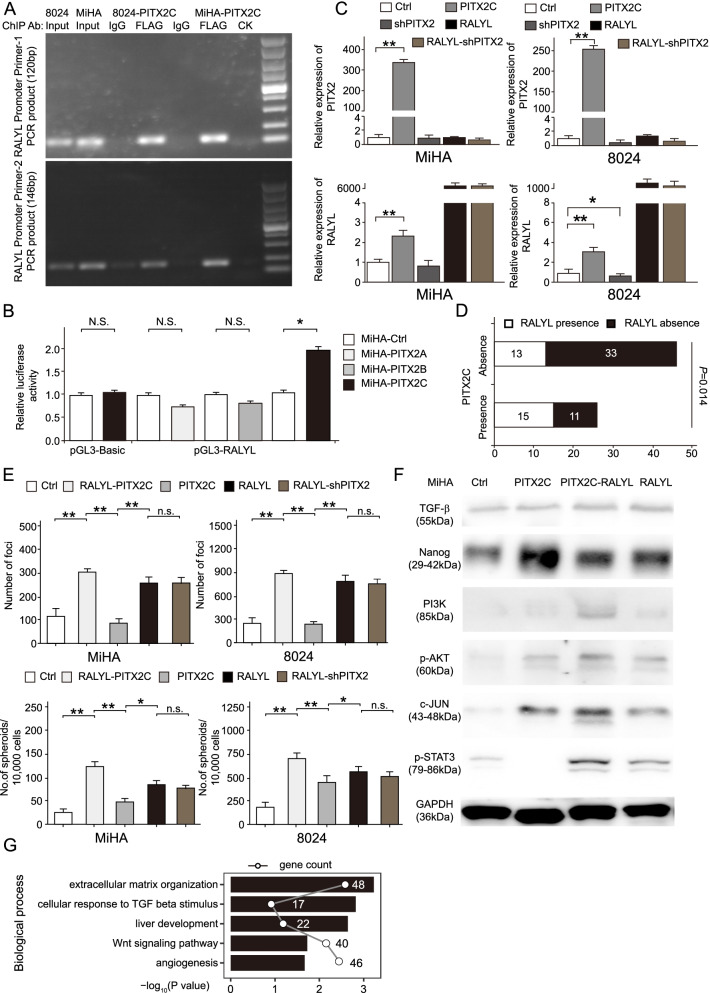
*PITX2C* enhances stemness through up-regulating the key developmental factors in liver progenitor. **A** ChIP-PCR results showing the binding of PITX2C at the promoter regions of *RALYL*. **B** The promoter region of RALYL was fused with firefly luciferase reporter. The luciferase activity was augmented in *PITX2C* transfected MIHA cells. The values represent the mean ± SD of three independent experiments (*P < 0.05, two-sided Student’s *t*-test). **C** Summary bar chart of relative mRNA expression of PITX2 (top) and RALYL (bottom) in the corresponding transfected cells. Parental cell was used as control. **D** Association of *PITX2C* expression with RALYL expression in HCC clinical samples (Cohort-1). The relative expression levels of *PITX2C* or *RALYL* in HCC clinical samples were evaluated by ΔΔCt _*PITX2C/RALYL*_ = (Ct _*PITX2C/RALYL in tumor*_ – Ct _*GAPDH in tumor*_)-20 as the *PITX2C* and *RALYL* expression was almost absent in non-tumor tissue. The expression of *PITX2C* or *RALYL* was defined as presence in HCC cases in which ΔΔCt _*PITX2C/RALYL*_ < 0. The *P* value was calculated using Pearson Chi-square test. **E** Quantitative analyses of foci numbers (top) or spheroids (bottom) of each transfected group are shown in bar chart. Values indicate the mean ± SD of three independent experiments (*P < 0.05, **P < 0.01, independent Student’s *t*-test). Ctrl: control group; PITX2: *PITX2C*-transfected group; RALYL: *RALYL*-transfected group; PITX2-RALYL: both *PITX2C* and *RALYL*-transfected group; RALYL-shPITX2: RALYL and shPITX2-transfected group. **F** Western blotting was performed to determine the expression of TGF-β2, NANOG, PI3K, phosphorylated-AKT (p-AKT), p-STAT3, and c-Jun in the indicated cell lysates. **G** Biological process identified by DAVID gene ontology analysis

To characterize the downstream targets regulated by *PITX2C* in HCC, ChIP assay was carried out using anti-flag antibody in PITX2C-flag transfected PLC-8024 and MiHA cells. Primers were designed to selectively amplify the promoters of genes that are specifically highly expressed in the LP (Supplementary Fig. 1A). ChIP-PCR revealed PITX2C could bind to the promoter region of RALY RNA binding protein like (RALYL) (Fig. 5A). In the pGL3-promoter dual luciferase reporter system, the overexpression of *PITX2C* rather than *PITX2A/B* significantly enhanced the luminescent signals in the *PITX2C* transfected groups compared with the control groups (Fig. 5B). Our previous study confirmed that RALYL is a liver progenitor specific gene that enhances the stemness of HCC [26]. *PITX2C* overexpression or silencing increased or decreased the expression levels of *RALYL* respectively (Fig. 5C). Furthermore, the presence of *RALYL* was associated with that of *PITX2C* in 72 paired HCC clinical samples (*P* = 0.014, Pearson Chi-square test, Fig. 5D). The co-transfection of *PITX2C* and *RALYL* promoted cell growth and self-renewal in PLC-8024 and MiHA (Fig. 5E). Surprisingly, compared to cells transfected with *PITX2C* alone, *RALYL*-transfected cells showed stronger stemness, which may be due to the significantly higher levels of *RALYL* expression caused by its own transfection than by *PITX2C* overexpression. The expression level of *RALYL* was relatively low compared to *PITX2* expression in LP, and was almost absent in mature hepatocytes (Supplementary Fig. 1A) [26]. We speculated that RALYL expression could trigger stemness-related abilities in HCC even at very low level. Moreover, PITX2 was further silenced in *RALYL* overexpression system. However, the self-renewal abilities of cells were not significantly decreased (Fig. 5E). The exogenous expression of RALYL is so powerful to maintain the self-renew of HCC cells that PITX2 silencing failed to reverse the effects. As *RALYL* could sustain the stability of *TGF-β2* mRNA, *PITX2C* transfection also increased the expression levels of TGF-β signaling related targets, such as p-AKT, p-STAT3 and c-Jun (Fig. 5F).

To further investigate the whole genomic binding signature of PITX2C in HCC, high-throughput sequencing of ChIP products of PITX2C was performed. From the sequence results, high-score PITX2C binding motifs were identified. Interestingly, PITX2C shared similar binding motifs with key transcriptional factors in liver development, suggesting that PITX2C may cooperate with these transcriptional factors (e.g., FOXA1, FOXP1, HNF4A, LEF1, GATA3) to regulate hepatic differentiation (Supplementary Fig. 5A). Top enriched gene ontology (GO) analysis further showed that PITX2C binding targets participated in biological processes, including extracellular matrix organization, liver development, angiogenesis, TGF-β, and Wnt signaling (Fig. 5G and Supplementary Table 2). Moreover, PITX2C binding sites were found at the promoters of genes that regulated the expression of hepatic genes and were specifically expressed in the LP and PH stages (Supplementary Fig. 5B-C). These results further support the notion that PITX2C transcriptionally regulates and cooperates with the key factors in liver development.

### *PITX2* regulates the Wnt pathway in HCC

**Fig. 6:**
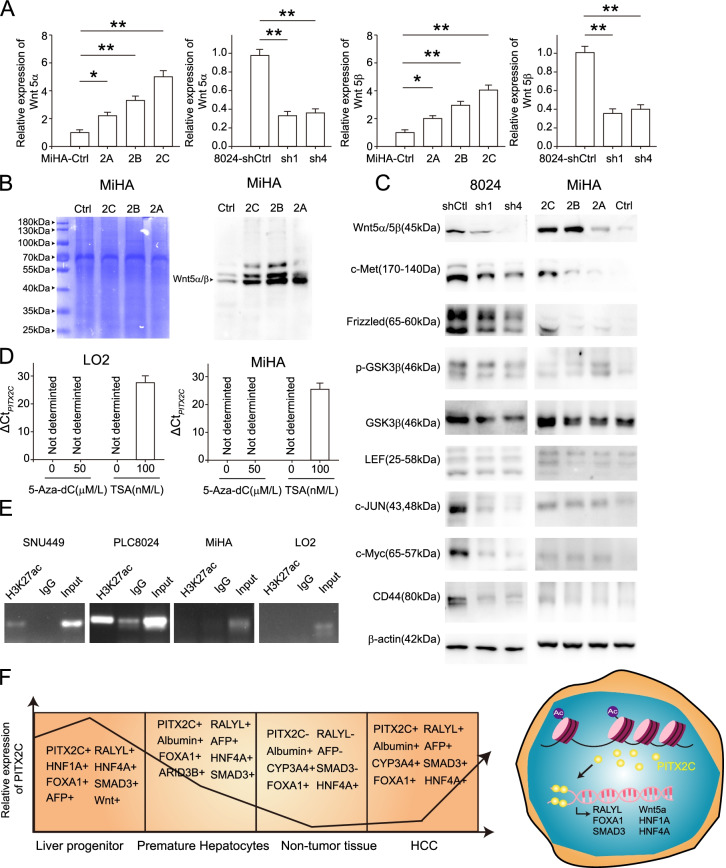
H3K27 acetylation in PITX2C promoter region and activation of the Wnt pathway by PITX2. **A** Relative expression of Wnt5α/β detected by qPCR in transfected cells compared with control cells (*P < 0.05, **P < 0.01, Student’s *t* test). **B** The Wnt5α/β secretion level in the cell culture medium was confirmed by western blotting. Total proteins staining with Coomassie Brilliant Blue were used as a loading control. **C** Western blotting was performed to determine the expression of key components of the Wnt pathway (Wnt5α/β, c-Met, Frizzled, p-GSK3b, GSK3b, LEF, c-JUN, c-Myc and CD44) in cell lysates from PLC-8024 and MiHA cells. β-Actin was used as a loading control. **D** Detection of *PITX2C* expression in LO2 and MiHA cells by qRT-PCR after demethylation (5-Aza-dC) and histone acetylation (trichostatin A) treatment. *PITX2C* expression can be detected when ΔCt_*PITX2C*_ (referred to Ct_*PITX2C*_-Ct_*GAPDH*_) < 30. **E** The enrichment of H3K27ac at the promoter regions of *PITX2C* was determined by ChIP-PCR with anti-H3K27ac antibody in LO2, MiHA, PLC-8024 and 449 cells. **F** The diagram on the left shows the dynamic expression of key markers and transcription factors during liver development and HCC progression. The diagram on the right illustrates the proposed mechanism of PITX2C upregulation in HCC differentiation and progression

PITX2 has been reported to interact with canonical and non-canonical Wnt pathways during development. The Wnt signaling cascade controls organogenesis by inducing a wide range of responses for cell proliferation and terminal differentiation during liver development [33]. To confirm the crosstalk between PITX2 and the Wnt pathway in HCC, the correlation between PITX2 and Wnt family members was analyzed using GEPIA [34]. Among the Wnt family members, the expression of *PITX2* was positively correlated with that of *Wnt5α* (Supplementary Fig. 5D). The overexpression of *PITX2A/B/C* or silencing of *PITX2* enhanced or reduced the expression levels of Wnt5α and Wnt5β, respectively (Fig. 6A). Consistently, western blotting showed that Wnt5α/5β secretion in cell culture supernatants was increased in *PITX2A/B/C*-overexpressing cells (Fig. 6B). Furthermore, *PITX2A/B/C* overexpression also up-regulated the protein levels of key components of the Wnt signaling pathway, including c-Met, LEF, Frizzled, p-GSK3β and its downstream targets, such as c-JUN, c-Myc and CD44. PITX2 silencing had the opposite effect (Fig. 6C). Taken together, our results demonstrate that PITX2 mediates the Wnt pathway and regulate sequential transcriptional and post-transcriptional events in this pathway.

### *PITX2C* expression is modulated by H3K27 acetylation in its promoter region

Three major PITX2 isoforms were generated by alternative splicing and the use of different promoters. PITX2A/B is transcribed using the same promoter region, however, PITX2C employs an alternative promoter located upstream of exon 4 [15]. In contrast to PITX2A/B, PITX2C is expressed at very low levels in HCC, as well as in mature hepatocytes. However, it was also clearly expressed in LP (Supplementary Fig. 1A). Low gene expression is often associated with aberrant methylation and histone deacetylation in promoter regions. Two cell lines, LO2 and MiHA with absent PITX2C, were treated with 5-Aza-dC, a DNA methyltransferase inhibitor or trichostatin A, a histone acetylation agent to investigate the effects of DNA methylation and histone acetylation on PITX2C expression. As shown in Fig. 6D, qRT-PCR analysis showed that *PITX2C* expression could be detected with 100 μM TSA, while it was not significantly affected by 5-Aza-dC treatment. These results imply that histone modification rather than aberrant promoter methylation is responsible for the low expression of *PITX2C* in HCC and hepatocytes. ChIP assays using H3K27ac antibody in LO2, MiHA, PLC-8024 and SNU-449 cells were performed. Thereafter, the ChIP products were amplified using specific primers targeting the upstream region of *PITX2C*. The enrichment of H3K27ac at the promoter of *PITX2C* was identified in PLC-8024 and SNU-449 cells, which expressed *PITX2C* at relatively higher levels, whereas weak H3K27 acetylation was observed in LO2 and MiHA cells (Fig. 6E). These data suggested that the expression of *PITX2C* be modulated by H3K27 acetylation in different hepatocytes.

## Discussion

Emerging evidence suggests that the malignant transformation of tumors is associated with the reactivation features of tissue progenitor cells [35]. In liver development, lineage-specific factors often undergo a period of activation, thereafter gradually decreasing until terminal differentiation. The expression levels of some lineage-specific factors (eg. AFP, CD133, Lgr5 and RALYL) are almost absent in mature hepatocytes [26]. HCC exhibits a similar gene expression pattern to that of LPs. Likewise, the epithelial-to-mesenchymal transition (EMT) plays an important role in liver bud morphogenesis as LPs delaminate and migrate into the septum transversum mesenchyme [4]. Liver lineage-specific factors confer progenitor-like features and contribute to the progression of HCC [36]. To investigate the unexplored roles of liver progenitor-specific factors in HCC, we established an in vitro hepatocyte differentiation model [10, 26, 28]. By the transcriptome sequencing of cells at four stages of this model and HCC clinical samples, PITX2 was identified. Previously, studies have reported that PITX2 is highly expressed in the fetal liver at embryonic day 9.5 and activated in the adult liver at 24 h after partial hepatectomy, during which LPs are stimulated to initiate liver regeneration [37, 38]. To identify the PITX2 isoform that contributes to liver differentiation, the qPCR targeting specific sequences of the three isoforms was performed. As a result, we found that *PITX2C*, which is highly expressed in liver progenitors, decreased in premature and normal hepatocytes, but was reactivated in HCC.

PITX2A/B/C has distinct functions in the tumorigenicity of HCC. PITX2C rather than PITX2A/B enhanced both the size and number of primary and secondary spheroids formed by PLC-8024 and MiHA cells. In addition, the overexpression of *PITX2C* up-regulated the markers of liver progenitors, such as *afp* and *lgr5*. Various functions of PITX2 isoforms in tumorigenicity may be responsible for its controversial role in different cancers [19–25, 39]. To characterize the role of PITX2 in the development and progression of cancer, there is a need to confirm the expression level of each PITX2 isoform at first.

Tumor lineage plasticity is considered to be one of the main causes of therapeutic resistance and recurrence. The feature commonly shared between tumors with poor differentiation is the re-expression of specific markers in progenitor cells, which usually have low expression, even completely absent in normal terminal differentiated cells. Among the selected nuclear proteins in our study, PITX2 was predicted to interact or regulate other factors by Pathway Commons. PITX2 has been widely reported to be significantly involved in regulating the Wnt and non-canonical pathways [13, 24, 33, 40, 41]. Similar to previous studies, our study confirmed the role of PITX2A/B/C in the Wnt signaling pathway by promoting Wnt5α/5β secretion in HCC. We further found that only PITX2C could bind to the promoter region of RALYL, which was identified as a stemness-related factor in HCC, as well as maintaining the mRNA stability of TGF-β, while PITX2A/B did not possess this capacity. In addition, both TGF-β and Wnt signaling were enriched in the ChIP-seq of PITX2C. Evidence suggests that signals from the TGF-β and Wnt pathways regulate hepatocyte and biliary epithelium differentiation [4]. Further analysis of ChIP-seq showed that PITX2C shared similar binding sites of liver-enriched transcription factors (FOXA1, HNF1A and HNF4A) which were reported to regulate liver-specific transcripts, suggesting that PITX2C cooperates with them during liver development. Moreover, PITX2C could bind to the promoter regions of these factors, which is in line with these factors functioning in a complex inter-regulatory network (Fig. 6F) [42–44].

## Conclusion

The findings presented in this study suggest PITX2C is a newly discovered and important factor in hepatic differentiation. The characterization of PITX2C could provide a basis for the development of HCC therapeutic strategies targeting specific factors in LPs.

## Supplementary Information

**Additional file 1: Supplementary Materials and Methods**. **Supplementary Figure 1.** PITX2 selection. (A) Heatmap of the expression profiles of the selected genes which were highly expressed in LP and PH. These genes included the specific genes for LP cells (*AFP*, *GATA3*, *NPNT*, *FOXA1*, *SMAD3*, *FOXF1*, *CDX2*) and the others encoding nuclear protein which showed a similar expression pattern to LP markers. (B) Among those selected nuclear protein genes, PITX2 was located in the central of gene regulatory network (Pathway Common). (C) Screenshot from SeqMan browser (Lasergene software 7.0) showing the varing 5′ sequences of the full-length of PIT2XA/B/C (*PITX2-V1 and V6*:PITX2A; *PITX2-V2, V4 and V5*:PITX2B: *PITX2-V3*: PITX2C). (D) Western blotting analysis confirmed the protein levels of PITX2 in immortalized liver cells and HCC cell lines. GAPDH was used as a loading control. (E) Representative images of FISH staining of *PITX2C* (red) in HCC cases with low, moderate and relative high expression levels of PITX2C. DAPI (blue) was used for nuclei counterstaining. (F) Kaplan-Meier overall (left) and disease-free (right) survival curve of two HCC groups in TCGA cohort: PITX2 (+), patients with higher PITX2 expression; PITX2 (−), patients with lower PITX2 expression. **Supplementary Figure 2.** PITX2A/B/C has distinct function in the tumorigenicity of HCC. Representative images of foci formation assay (A) and colony formation (B) in *PITX2A/B/C*-transfected cells and control cells. (C) Two shRNAs targeting PITX2 (shPITX2–1 and shPITX2–4) effectively decreased the mRNA level of PITX2 in PLC-8024 and SNU449 detected by qRT-PCR. Non-transfected cells were used as controls. Data are presented as the mean ± SD of 3 independent experiments. (*P < 0.05, **P < 0.01, independent Student’s *t*-test) (D) The cell proliferation between shPITX2 -transfected cells and control cells was compared by XTT assay. The results are expressed as the mean ± SD of three independent experiments. (*P < 0.05, **P < 0.01, independent Student’s *t*-test). Representative images (left) and summary bar chart (right) of foci formation assay (E) and colony formation in soft agar assay (F) in shPITX2-transfected and control cells. Values indicate the mean ± SD of 3 independent experiments (*P < 0.05; **P < 0.01; independent Student *t* test). (G) Orthotopic tumor formation was performed via intrahepatic implantation experiments using *PITX2A*-transfected cells and control cells or shPITX2-transfected cells and control cells. The final tumor volumes are summarized in the dot chart. Average tumor volume is expressed as the mean ± SD of mice. The *P* value was calculated using paired Student’s *t* test. (H) Representative images of excised orthotopic tumor formed by intrahepatic implantation experiment using *PITX2A-*transfected Hep3B cells and control cells. **Supplementary Figure 3.** PITX2C promotes cell mobility, self-renewal and chemoresistance of HCC. (A) Representative images (top) and bar chart (bottom) of cell migration and invasion abilities in s*hPITX2*-transfected and control cells by Transwell and Matrigel invasion assays. Migrated and invaded cells were stained with crystal violet and counted under a microscope. Values indicate the mean ± SD of three independent experiments (*P < 0.05; **P < 0.01; independent Student *t* test). (B) Representative images of spheroid formation assay using *shPITX2-*transfected cells and control cells (left). The numbers of primary and secondary spheroids are calculated in the bar chart (right). Values indicate the mean ± SD of three independent experiments (*P < 0.05, **P < 0.01, independent Student’s *t*-test). The apoptotic indexes of *PITX2C*-transfected (C), *shPITX2*-transfected cells (D) and control cells were detected by fluorescence-activated cell sorting-based Annexin V/AAD double staining after treatment with 5-Fu or Sorafenib at the indicated concentrations for 48 h. (E) The mRNA levels of *AFP* and *Lgr5* were compared by ΔCt in *PITX2C* or *shPITX2*- transfected cells and control cells (ΔCt_*AFP*_ = Ct_*AFP*_-Ct_*GAPDH*_; ΔCt_*Lgr5*_ = Ct_*Lgr5*_-Ct_*GAPDH*_). **Supplementary Figure 4.** Representatives of IHC staining images with anti-EPCAM, CD133, c-Myc and NANOG in tumors induced by 8024-Ctrl, 8024-PITX2C cells with 5-FU treatment. Red arrows indicate cancer stem cells. **Supplementary Figure 5.** Analysis of the ChIP sequencing data. (A) PITX2C shared similar binding motifs with several key transcription factors in LP. (B) Screenshot from the WashU epigenome browser showing PITX2C binding sites at the promoter of *HNF1A*, *HNF4A*, *FOXA1*, *SMAD3*, and *ARID5B*. (C) Heatmap of the expression profile for *HNF4A*, *FOXA1*, *SMAD* and *ARID5B* in the four stages (ES, EN, LP, PH) of in vitro hepatocyte differentiation model. (D) The expression of PITX2 is positively correlated with that of Wnt5α in GEPIA. **Table S1.** List of PCR primers for PITX2A/B/C expression.

**Additional file 2: Table S2.** The gene ontology (GO) analysis of PITX2C ChIP-seq.

## Data Availability

The data sets used and/or analyzed during the current study are available from the corresponding author on reasonable request.

## Abbreviations

PITX2: Paired like Homeodomain 2
HCC: Hepatocellular carcinoma
AFP: Alpha fetoprotein
ES: Embryonic stem cells
DE: Definitive endoderm
LP: Liver progenitor
PH: Premature hepatocytes
5-Fu: 5-fluorouracil
qRT-PCR: Quantitative real-time PCR
Ct: Cycle threshold
ChIP: Chromatin immunoprecipitation
