# Supplementary material for: PITX2C increases the stemness features of hepatocellular carcinoma cells by up-regulating key developmental factors in liver progenitor
Source: J Exp Clin Cancer Res. 2022 Jun 28;41:211. doi: 10.1186/s13046-022-02424-z (PMC9238105; doi:10.1186/s13046-022-02424-z)
Supplement: Supplementary file 1 — Additional file 1: Supplementary Materials and Methods. Supplementary Figure 1. PITX2 selection. (A) Heatmap of the expression profiles of the selected genes which were highly expressed in LP and PH. These genes included the specific genes for LP cells (AFP, GATA3, NPNT, FOXA1, SMAD3, FOXF1, CDX2) and the others encoding nuclear protein which showed a similar expression pattern to LP markers. (B) Among those selected nuclear protein genes, PITX2 was located in the central of gene regulatory network (Pathway Common). (C) Screenshot from SeqMan browser (Lasergene software 7.0) showing the varing 5′ sequences of the full-length of PIT2XA/B/C (PITX2-V1 and V6:PITX2A; PITX2-V2, V4 and V5:PITX2B: PITX2-V3: PITX2C). (D) Western blotting analysis confirmed the protein levels of PITX2 in immortalized liver cells and HCC cell lines. GAPDH was used as a loading control. (E) Representative images of FISH staining of PITX2C (red) in HCC cases with low, moderate and relative high expression levels of PITX2C. DAPI (blue) was used for nuclei counterstaining. (F) Kaplan-Meier overall (left) and disease-free (right) survival curve of two HCC groups in TCGA cohort: PITX2 (+), patients with higher PITX2 expression; PITX2 (−), patients with lower PITX2 expression. Supplementary Figure 2. PITX2A/B/C has distinct function in the tumorigenicity of HCC. Representative images of foci formation assay (A) and colony formation (B) in PITX2A/B/C-transfected cells and control cells. (C) Two shRNAs targeting PITX2 (shPITX2–1 and shPITX2–4) effectively decreased the mRNA level of PITX2 in PLC-8024 and SNU449 detected by qRT-PCR. Non-transfected cells were used as controls. Data are presented as the mean ± SD of 3 independent experiments. (*P < 0.05, **P < 0.01, independent Student’s t-test) (D) The cell proliferation between shPITX2 -transfected cells and control cells was compared by XTT assay. The results are expressed as the mean ± SD of three independent experiments. (*P < 0.05, **P < 0.01, ind [file 13046_2022_2424_MOESM1_ESM.zip › Jiang et al.Clean Suppplementary Materials.docx]

**Supplementary Materials and Methods**

**Cell culture**

Cells were maintained in high-glucose Dulbecco’s modified Eagle medium (Gibco BRL, Grand Island, NY, USA) supplemented with 10% fetal bovine serum (FBS) (Gibco BRL) and 1% penicillin/streptomycin. The cells were incubated at 37 °C in a humidified incubator containing 5% CO_2_.

**Construction of PITX2A/B/C overexpression and knockdown cells**

To study the function of PITX2A/B/C individually, full-length coding sequences of human *PITX2A/B/C* clones were purchased from Shanghai Zorin Biotechnology Co., Ltd (Shanghai, China) and cloned into pLenO-GTP-C-3×flag vector. For the PITX2 knockdown assay, two short hairpin RNAs (shRNAs) specifically targeting *PITX2* were cloned into the PLL3.7 lentiviral vector (Addgene). The sequence of shPITX2-1 is 5’-GATGCAATGATGTTTCTGAAA-3’ and shPITX2-4 is

5’- ATGTTATACAGAAGCGATTAG-3’. Puromycin (Sigma-Aldrich, St.Louis, MO, USA) was used to select stably transduced cells.

**RNA extraction and quantitative real-time PCR (qRT-PCR)**

Total RNA was extracted using RNeasy Plus Universal Kits (Qiagen), and reverse transcription was performed using a reverse-transcription PCR kit (Roche, Basel, Switzerland), according to the manufacturer’s instructions. qRT-PCR was performed using SYBR Green PCR master mix (Roche) on an ABI Prism 7900HT system with specific primers (Supplementary Table 1). All qRT-PCR reactions were performed in triplicates.

**In *vitro* functional assays and animals**

In *vitro* tumorigenicity was assessed by XTT cell proliferation (Roche Diagnostics, Indianapolis, IN), foci formation and colony formation in soft agar. Cell motility was assessed by cell migration and invasion arrays using Transwell chambers with or without Matrigel (BD Biosciences, CA, USA). A spheroid formation assay was performed to assess the self-renewal ability. HCC patients derived organoid establishment was reported in our previous paper[^1^](#_ENREF_1)^,^ [^2^](#_ENREF_2)^,^ [^3^](#_ENREF_3). Intrahepatic tumor implantation experiments were performed to evaluate tumorigenicity in *vivo*[*^4^*](#_ENREF_4). A nude mouse xenograft model was used to evaluate chemoresistant ability[^1^](#_ENREF_1). In Brief, 3×10^6^ PLC-8024 cells with PITX2C overexpression or PLC-8024 control cells were subcutaneously injected into the dorsal flank of 4-5 week old BALB/cAnN-nu (nude) mice. As *in vitro* functional assays showed PITX2C may inhibit cell proliferation, PLC-8024 cells with PITX2C overexpression were injected into nude mice 1-2 weeks prior to control cells injection to acquire the subcutaneous tumors with similar size. After 4-6 weeks, we selected 6 mice per group which bore subcutaneous tumor with similar size of approximately 5 mm in diameter for the following 5-FU treatment. 5-FU was administrated via intraperitoneal injection every five days. All experiments conducted on animals were approved by the University of Hong Kong Committee on the Use of Live Animals in Teaching and Research (CULATR).

**Immunohistochemical staining (IHC)**

Paraffin-embedded tissue sections were deparaffinized and rehydrated. Slides were immersed in 10 mM citrate buffer, boiled for 15 min in a microwave oven, and incubated with primary antibody at 4 °C overnight in a moist chamber, and sequentially incubated with biotinylated general secondary antibody for 1 h at room temperature, followed by streptavidin–peroxidase conjugate for 15 min at room temperature. Finally, a 3, 5-diaminobenzidine substrate kit (Dako) was used for color development followed by Mayer’s hematoxylin counterstaining.

**Western blotting analysis and antibodies**

Western blotting analysis was performed according to the standard procedure. Mouse antibodies for β-actin, GAPDH, rabbit antibodies for N-cadherin, E-cadherin, α-catenin, β-catenin, vimentin, PARP, cleaved-caspase 9, caspase-9, PI3K, p-AKT, c-JUN, p-STAT3, Wnt5α/5β, Frizzled, LEF, c-Myc, NANOG, CD44, CD133 and EPCAM were purchased from Cell signaling Technology (Danvers, MA, USA). p-GSK3β and GSK3β were purchased from Proteintech (Rosemont, IL, USA). PITX2 was purchased from ABclonal Biotechnology Co., Ltd (Wuhan, China).

**Fluorescence *in situ* hybridization (FISH)**

Cells were fixed and permeabilized in PBS containing 0.5% Triton X-100. Cy3 labeled FISH probes were designed by RiboBio (Guangzhou, China). Hybridization was carried out overnight in a humidified chamber at 37 °C in the dark. After counterstaining with DAPI (Beyotime, Shanghai, China), the slides were visualized under a Leica SP8 X confocal microscope (Leica, Germany).

**Chemotherapy-induced cytotoxicity and apoptotic assay**

Drugs-induced cytotoxicity (5-Fu and sorafenib) was determined using the XTT cell proliferation assay according to the manufacturer’s instructions. The apoptotic assay was performed using flow cytometry. After treating with 5-Fu or sorafenib for 48 h, cells were harvested and double stained with PE-conjugated Annexin-V and 7-aminoactinomycin (7-AAD) provided by the BD apoptosis detection kit (BD Biosciences, San Jose, CA). FlowJo (Version 10.0, Tree Star) and the FACS Canto II Analyzer (BD Biosciences) were used for analysis.

**Luciferase reporter assays**

The lucifearse reporter plasmid was cloned by inserting the promoter region of RALYL. Cells seeded in 96-well plates were transfected with 100 ng of reporter plasmid. After 72 h, the cells were analyzed using the Dual-Glo Luciferase system (Promega). The Renilla luciferase plasmid was used to normalize the transfection efficiency of the reporter plasmid.

**Chromatin immunoprecipitation**

Briefly, 2×10^6^ fixed cells were lysed to prepare the nuclear extracts for each sample. After chromatin shearing by sonication, lysates were incubated overnight at 4°C with protein A Dynabeads (Invitrogen, USA) coupled with 5-10 μg of antibody. After immunoprecipitation, the beads were recovered using a magnet and then washed. ChIP DNA was eluted and crosslinks reverted at 55℃ for 2 h then purified with the QIAquick PCR Purification Kit (Qiagen, USA). DNA was quantitated using the Qubit dsDNA HS assay and a Qubit3.0 Fluorimeter (Invitrogen). For ChIP-seq, 5-10 ng of purified DNA was used to generate the sequencing library using a VAHTS Universal DNA Library Prep Kit for Illumina V3 (Vazyme Biotech, China) and sequenced with Illumina HiSeq X Ten with 150 bp paired-end reads (Novogene Biotech, China). For ChIP-seq, 5-10 ng of purified DNA was used to generate the sequencing library using a VAHTS Universal DNA Library Prep Kit for Illumina V3 (Vazyme Biotech, China) and sequenced with Illumina HiSeq X Ten with 150 bp paired-end reads (Novogene Biotech, China).

**ChIP-seq data processing**

Quality control of ChIP-seq data was accomplished using FastQC (http://www.bioinformatics.babraham.ac.uk/projects/fastqc/). All raw sequencing reads were trimmed and aligned to the hg19 genome using Bowtie2[^5^](#_ENREF_5). Duplicate reads were removed with SAMtools[^6^](#_ENREF_6), and MACS2[^7^](#_ENREF_7) was used to identify the genomic regions of ChIP-seq peaks with default parameters. For the normalization and visualization of the sequencing data, deepTools 2.0 [^8^](#_ENREF_8)was used to generate bigWig files. The genomic signal tracks were visualized using the WashU Epigenome Browser (<https://epgg-test.wustl.edu/browser/>). Pathway enrichment analysis was performed for the differentially expressed genes using Database for Annotation, Visualization and Integrated Discovery (DAVID).

**Supplementary Figure Legend**

**Supplementary Figure 1. PITX2 selection. (A)** Heatmap of the expression profiles of the selected genes which were highly expressed in LP and PH. These genes included the specific genes for LP cells (*AFP*, *GATA3*, *NPNT*, *FOXA1*, *SMAD3*, *FOXF1*, *CDX2*) and the others encoding nuclear protein which showed a similar expression pattern to LP markers. **(B)** Among those selected nuclear protein genes, PITX2 was located in the central of gene regulatory network (Pathway Common). **(C)** Screenshot from SeqMan browser (Lasergene software 7.0) showing the varing 5’ sequences of the full-length of PIT2XA/B/C (*PITX2-V1 and V6*：PITX2A; *PITX2-V2, V4 and V5*：PITX2B: *PITX2-V3*: PITX2C ). **(D)** Western blotting analysis confirmed the protein levels of PITX2 in immortalized liver cells and HCC cell lines. GAPDH was used as a loading control. **(E)** Representative images of FISH staining of

*PITX2C* (red) in HCC cases with low, moderate and relative high expression levels of PITX2C. DAPI (blue) was used for nuclei counterstaining. **(F)** Kaplan-Meier overall (left) and disease-free (right) survival curve of two HCC groups in TCGA cohort: PITX2 (+), patients with higher PITX2 expression; PITX2 (-), patients with lower PITX2 expression.

**Supplementary Figure 2. PITX2A/B/C has distinct function in the tumorigenicity of HCC.** Representative images of foci formation assay **(A)** and colony formation **(B)** in *PITX2A/B/C*-transfected cells and control cells. **(C)** Two shRNAs targeting PITX2 (shPITX2-1 and shPITX2-4) effectively decreased the mRNA level of PITX2 in PLC-8024 and SNU449 detected by qRT-PCR. Non-transfected cells were used as controls. Data are presented as the mean ± SD of 3 independent experiments.

(**P* < 0.05, ***P* < 0.01, independent Student’s *t*-test) **(D)** The cell proliferation between shPITX2 -transfected cells and control cells was compared by XTT assay. The results are expressed as the mean ± SD of three independent experiments. (**P* < 0.05, ***P* < 0.01, independent Student’s *t*-test). Representative images (left) and summary bar chart (right) of foci formation assay **(E)** and colony formation in soft agar assay **(F)** in shPITX2-transfected and control cells. Values indicate the mean ± SD of 3 independent experiments (**P* < 0.05; ***P* < 0.01; independent Student *t* test). **(G)** Orthotopic tumor formation was performed via intrahepatic implantation experiments using *PITX2A*-transfected cells and control cells or shPITX2-transfected cells and control cells. The final tumor volumes are summarized in the dot chart. Average tumor volume is expressed as the mean ± SD of mice. The *P* value was calculated using paired Student’s *t* test. **(H)** Representative images of excised orthotopic tumor formed by intrahepatic implantation experiment using *PITX2A-*transfected Hep3B cells and control cells.

**Supplementary Figure 3. PITX2C promotes cell mobility, self-renewal and chemoresistance of HCC. (A)** Representative images (top) and bar chart (bottom) of cell migration and invasion abilities in s*hPITX2*-transfected and control cells by Transwell and Matrigel invasion assays. Migrated and invaded cells were stained with crystal violet and counted under a microscope. Values indicate the mean ± SD of three independent experiments (**P* < 0.05; ***P* < 0.01; independent Student *t* test). **(B)** Representative images of spheroid formation assay using *shPITX2-*transfected cells and control cells (left). The numbers of primary and secondary spheroids are calculated in the bar chart (right). Values indicate the mean ± SD of three independent experiments (**P* < 0.05, ***P* < 0.01, independent Student’s *t*-test). The apoptotic indexes of *PITX2C*-transfected **(C)**, *shPITX2*-transfected cells **(D)** and control cells were detected by fluorescence-activated cell sorting-based Annexin V/AAD double staining after treatment with 5-Fu or Sorafenib at the indicated concentrations for 48h. **(E)** The mRNA levels of *AFP* and *Lgr5* were compared by ΔCt in *PITX2C* or *shPITX2*- transfected cells and control cells (ΔCt*_AFP_* =Ct*_AFP_*-Ct*_GAPDH_*; ΔCt*_Lgr5_* =Ct*_Lgr5_*-Ct*_GAPDH_*)

**Supplementary Figure 4.** Representatives of IHC staining images with anti-EPCAM, CD133, c-Myc and NANOG in tumors induced by 8024-Ctrl, 8024-PITX2C cells with 5-FU treatment. Red arrows indicate cancer stem cells.

**Supplementary Figure 5. Analysis of the ChIP sequencing data. (A)** PITX2C shared similar binding motifs with several key transcription factors in LP. **(B)** Screenshot from the WashU epigenome browser showing PITX2C binding sites at the promoter of *HNF1A*, *HNF4A*, *FOXA1*, *SMAD3*, and *ARID5B*. **(C)** Heatmap of the expression profile for *HNF4A*, *FOXA1*, *SMAD* and *ARID5B* in the four stages (ES, EN, LP, PH) of *in vitro* hepatocyte differentiation model. **(D)** The expression of PITX2 is positively correlated with that of Wnt5α in GEPIA.

**Table S1.** List of PCR primers for PITX2A/B/C expression

| Primer | Sequence(5’ → 3’) |
| --- | --- |
| PITX2-F | GCCAAGGGCCTTACATCCG |
| PITX2-R | GGTGGGGAAAACATGCTCT |
| PITX2-V16-F | ggagaccaactgccgcaaact |
| PITX2- V16-R | AGCGGTTCCTCTGGAAAGTG |
| PITX2-V245-F | TGAGAGCCGAAAAGAGGCAG |
| PITX2- V245-R | AGCGGTTCCTCTGGAAAGTG |
| PITX2-V3-F | gctggaggtgcacaccatctc |
| PITX2- V3-R | AGCGGTTCCTCTGGAAAGTG |
| GAPDH-F | GGAGCGAGATCCCTCCAAAAT |
| GAPDH-R | GGCTGTTGTCATACTTCTCATGG |
| RALYL-promoter-F1 | GAGAGGCTGTGCGTTCAGAT |
| RALYL-promoter-R1 | CCCTAGATCTGCGTTTCCCC |
| RALYL-promoter-F2 | AGAATCCACTGCTCAGCCAC |
| RALYL-promoter-R2 | GAGAGGGGCATGGGAAACAA |
| RALYL-F | GCGCCTGGAGAAGATTGAGA |
| RALYL-R | GAAACAGCTCATGACCCCCA |
| PITX2C-Promoter-F1 | TCTCGGGTTAGGACGAGCTT |
| PITX2C-Promoter-R2 | AGTCGCCTCAGATCACGTTC |
| Wnt5α-F | CTCCATTCCTGGGCGCATC |
| Wnt5α-R | ATCCCCAAAGCAACTCCTGG |
| Wnt5β-F | TCTTTGGCTCGGAAACGGTG |
| Wnt5β-R | TAATGACCACCAGGAGTTGGC |

**Supplementary Reference**

1. Wang X, Wang J, Tsui YM, Shi C, Wang Y, Zhang X*, et al.* RALYL increases hepatocellular carcinoma stemness by sustaining the mRNA stability of TGF-beta2. *Nature communications* 2021, **12**(1)**:** 1518.

2. Liu M, Yan Q, Sun Y, Nam Y, Hu L, Loong JH*, et al.* A hepatocyte differentiation model reveals two subtypes of liver cancer with different oncofetal properties and therapeutic targets. *Proceedings of the National Academy of Sciences of the United States of America* 2020, **117**(11)**:** 6103-6113.

3. Yan Q, Zhang Y, Fang X, Liu B, Wong TL, Gong L*, et al.* PGC7 promotes tumor oncogenic dedifferentiation through remodeling DNA methylation pattern for key developmental transcription factors. *Cell death and differentiation* 2021.

4. Jiang L, Yan Q, Fang S, Liu M, Li Y, Yuan YF*, et al.* Calcium-binding protein 39 promotes hepatocellular carcinoma growth and metastasis by activating extracellular signal-regulated kinase signaling pathway. *Hepatology* 2017, **66**(5)**:** 1529-1545.

5. Langmead B, Salzberg SL. Fast gapped-read alignment with Bowtie 2. *Nature methods* 2012, **9**(4)**:** 357-359.

6. Li H, Handsaker B, Wysoker A, Fennell T, Ruan J, Homer N*, et al.* The Sequence Alignment/Map format and SAMtools. *Bioinformatics* 2009, **25**(16)**:** 2078-2079.

7. Zhang Y, Liu T, Meyer CA, Eeckhoute J, Johnson DS, Bernstein BE*, et al.* Model-based analysis of ChIP-Seq (MACS). *Genome biology* 2008, **9**(9)**:** R137.

8. Ramirez F, Dundar F, Diehl S, Gruning BA, Manke T. deepTools: a flexible platform for exploring deep-sequencing data. *Nucleic acids research* 2014, **42**(Web Server issue)**:** W187-191.
