# Supplementary figures and images for: PITX2C increases the stemness features of hepatocellular carcinoma cells by up-regulating key developmental factors in liver progenitor
Source: J Exp Clin Cancer Res. 2022 Jun 28;41:211. doi: 10.1186/s13046-022-02424-z (PMC9238105; doi:10.1186/s13046-022-02424-z)

A

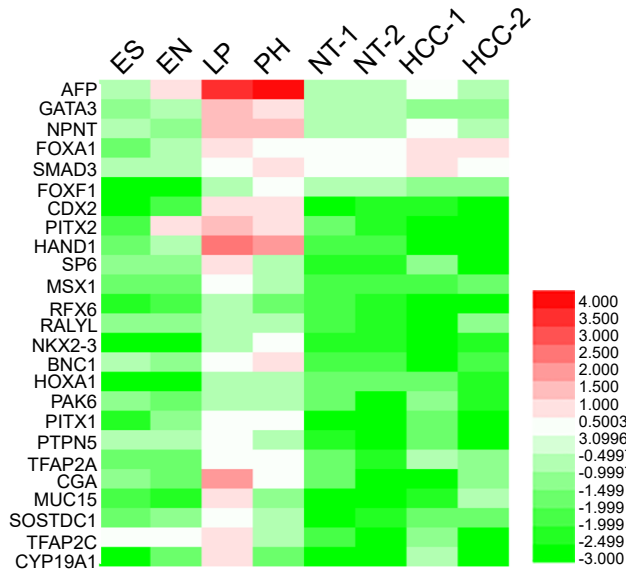

B

Figure S1

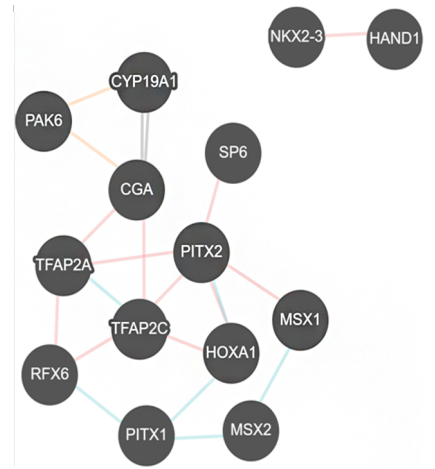

C

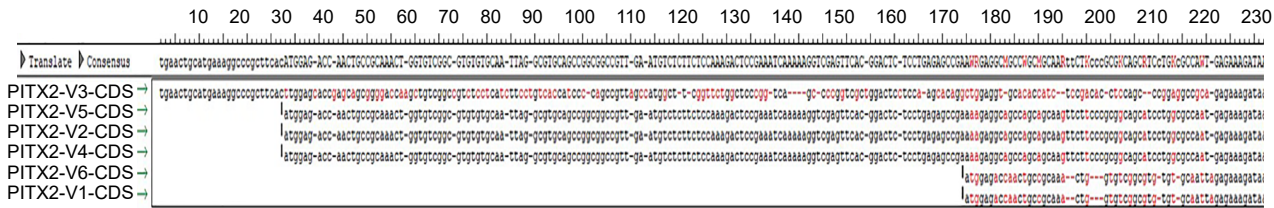

E

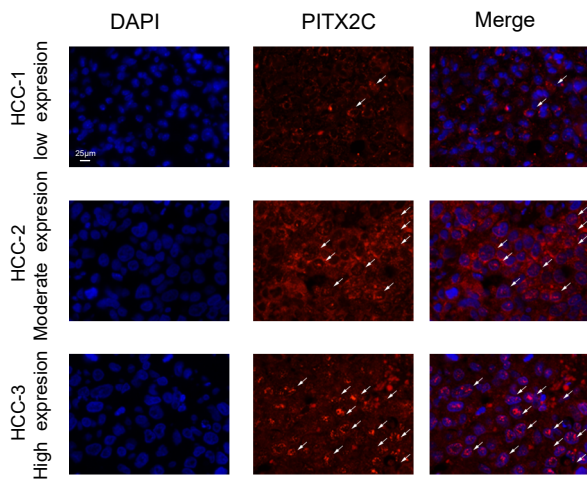

D

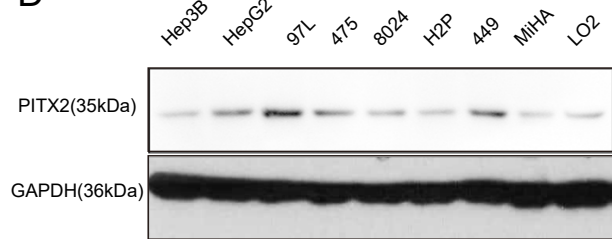

F

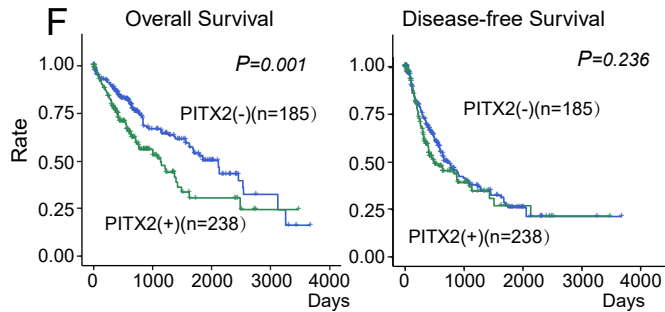

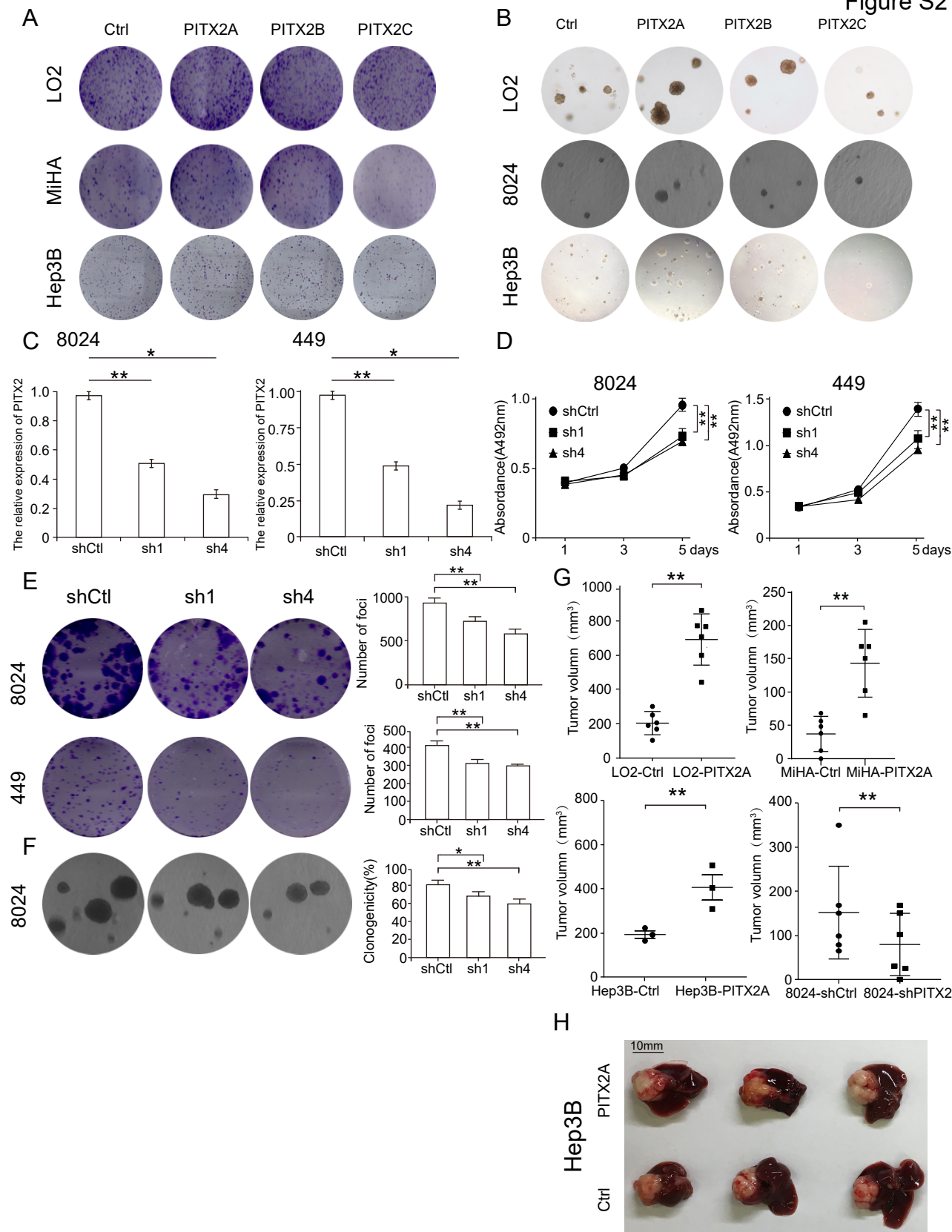

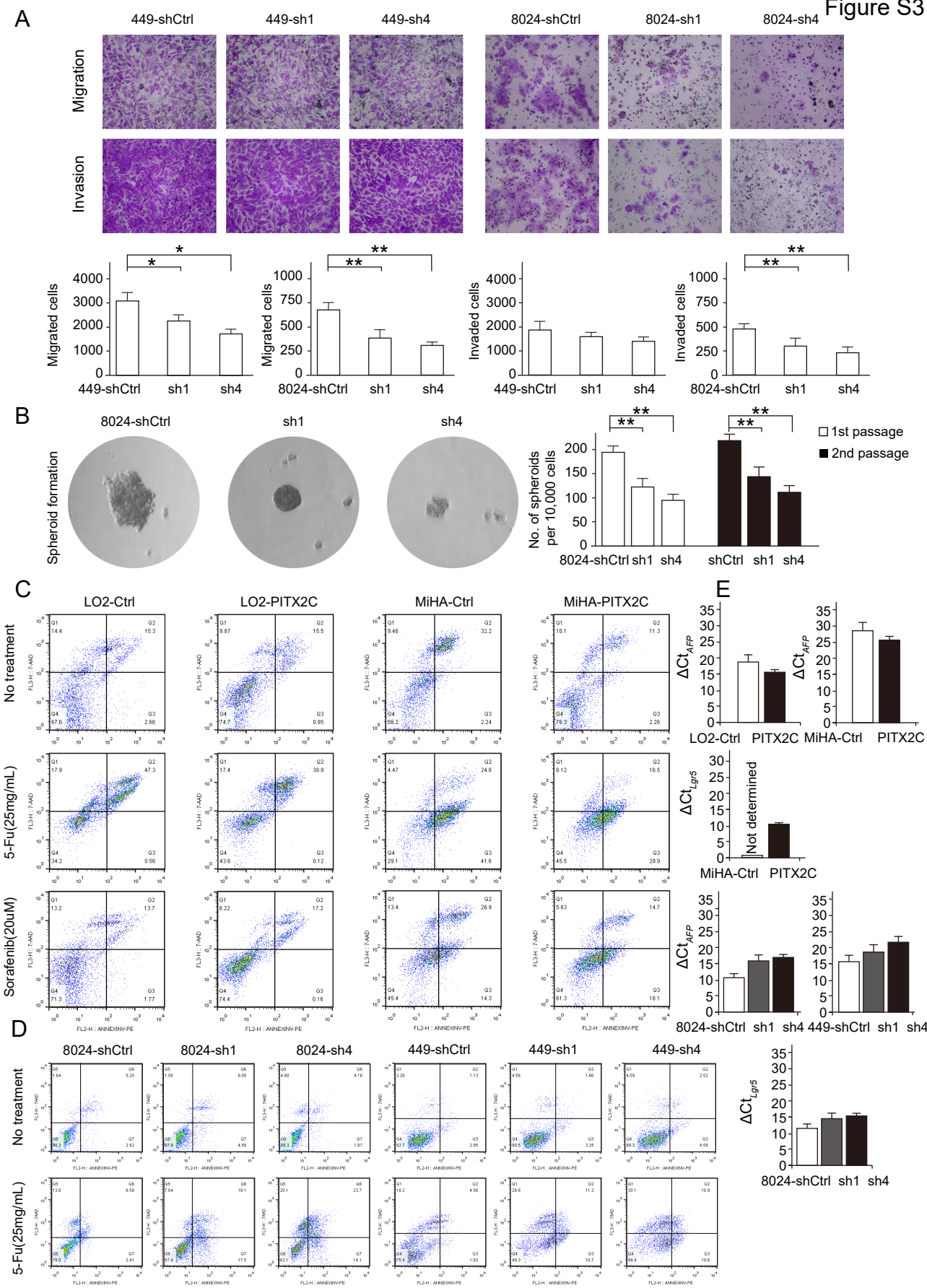

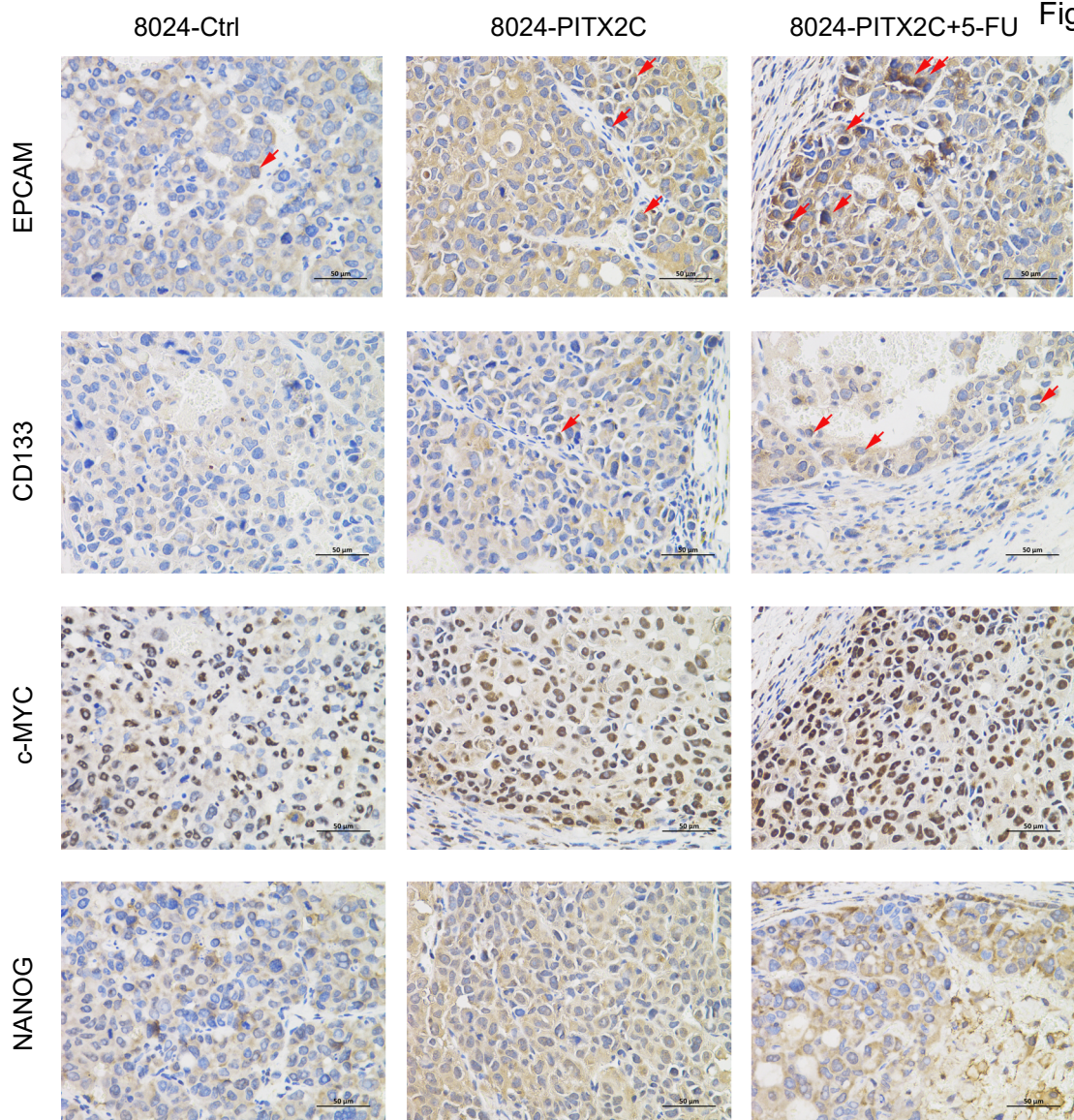

Figure S5

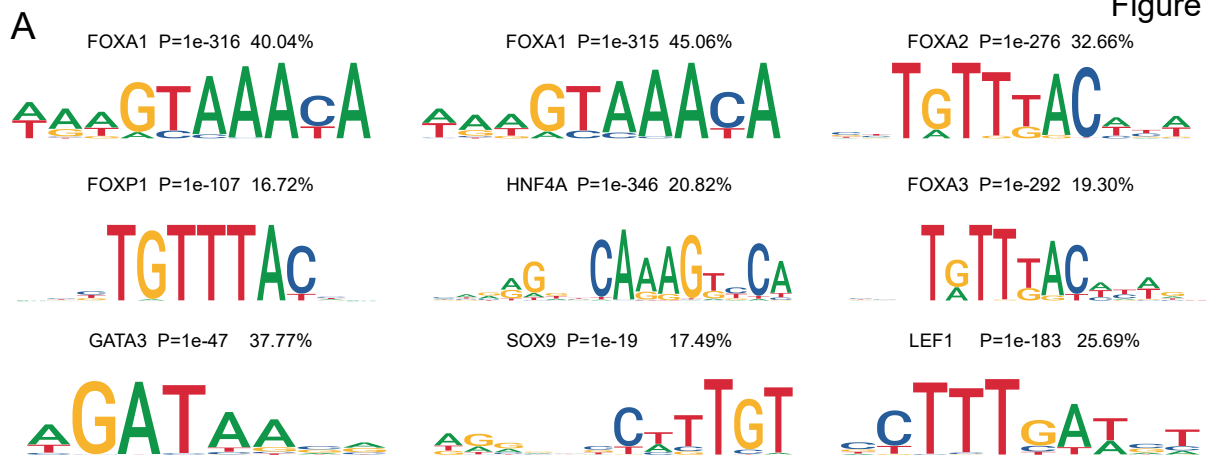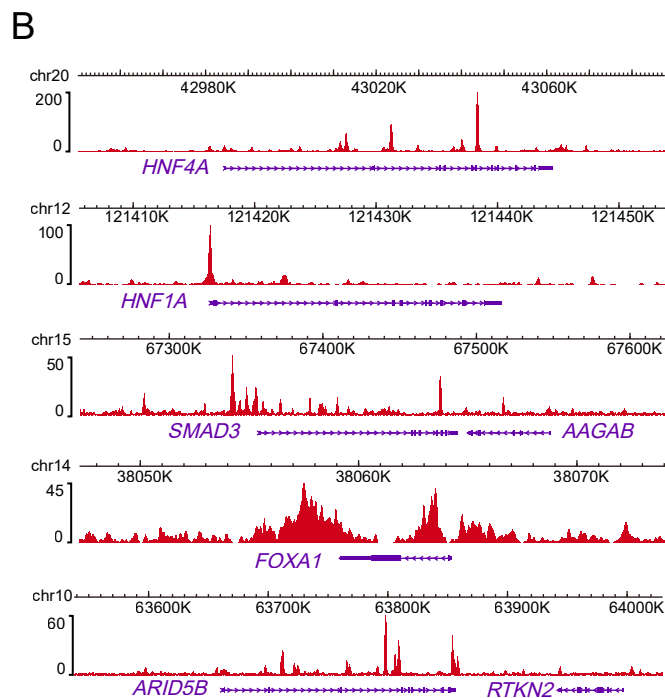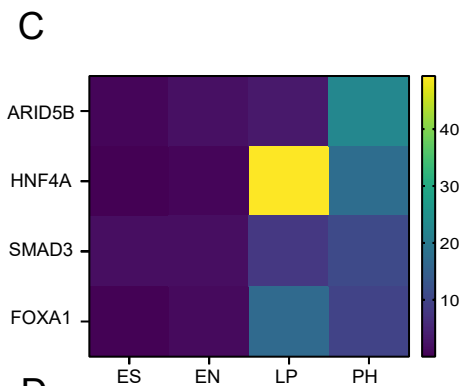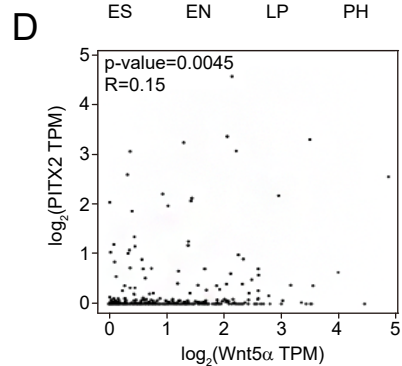

Supplement: Supplementary file 1 — Additional file 1: Supplementary Materials and Methods. Supplementary Figure 1. PITX2 selection. (A) Heatmap of the expression profiles of the selected genes which were highly expressed in LP and PH. These genes included the specific genes for LP cells (AFP, GATA3, NPNT, FOXA1, SMAD3, FOXF1, CDX2) and the others encoding nuclear protein which showed a similar expression pattern to LP markers. (B) Among those selected nuclear protein genes, PITX2 was located in the central of gene regulatory network (Pathway Common). (C) Screenshot from SeqMan browser (Lasergene software 7.0) showing the varing 5′ sequences of the full-length of PIT2XA/B/C (PITX2-V1 and V6:PITX2A; PITX2-V2, V4 and V5:PITX2B: PITX2-V3: PITX2C). (D) Western blotting analysis confirmed the protein levels of PITX2 in immortalized liver cells and HCC cell lines. GAPDH was used as a loading control. (E) Representative images of FISH staining of PITX2C (red) in HCC cases with low, moderate and relative high expression levels of PITX2C. DAPI (blue) was used for nuclei counterstaining. (F) Kaplan-Meier overall (left) and disease-free (right) survival curve of two HCC groups in TCGA cohort: PITX2 (+), patients with higher PITX2 expression; PITX2 (−), patients with lower PITX2 expression. Supplementary Figure 2. PITX2A/B/C has distinct function in the tumorigenicity of HCC. Representative images of foci formation assay (A) and colony formation (B) in PITX2A/B/C-transfected cells and control cells. (C) Two shRNAs targeting PITX2 (shPITX2–1 and shPITX2–4) effectively decreased the mRNA level of PITX2 in PLC-8024 and SNU449 detected by qRT-PCR. Non-transfected cells were used as controls. Data are presented as the mean ± SD of 3 independent experiments. (*P < 0.05, **P < 0.01, independent Student’s t-test) (D) The cell proliferation between shPITX2 -transfected cells and control cells was compared by XTT assay. The results are expressed as the mean ± SD of three independent experiments. (*P < 0.05, **P < 0.01, ind [file 13046_2022_2424_MOESM1_ESM.zip › Supplementary Figures.pdf]
